# Colorimetric Integrated PCR Protocol for Rapid Detection of *Vibrio parahaemolyticus*

**DOI:** 10.3390/s16101600

**Published:** 2016-09-28

**Authors:** Kewen Cheng, Daodong Pan, Jun Teng, Li Yao, Yingwang Ye, Feng Xue, Fan Xia, Wei Chen

**Affiliations:** 1 School of Marine Science, Ningbo University, Ningbo 315211, China; chengkewen100@163.com; 2 School of Food Science & Engineering, Hefei University of Technology, Hefei 230009, China; guanpeng0189@163.com (J.T.); ybl200999@sohu.com (L.Y.); yeyingwang04@126.com (Y.Y.); xuef1@jsciq.gov.cn (F.X.); 3 School of Chemistry & Chemical Engineering, Huazhong University of Science & Technology, Wuhan 430074, China; xiafan@hust.edu.cn

**Keywords:** bacterium detection, colorimetric, PCR, primer design, virulence gene

## Abstract

Rapid detection of pathogens is of great significance for food safety and disease diagnosis. A new colorimetric method for rapid and easy detection of *Vibrio parahaemolyticus* (*V. parahaemolyticus* or Vp) has been developed in this research. A specific sequence was designed and integrated with the forward primer for molecular detection of Vp. This specific sequence was tested and treated as the horseradish peroxidase (HRP)-mimicking DNAzyme and could be amplified during the polymerase chain reaction (PCR) process. The products of PCR including the sequence of HRP-mimicking DNAzyme could produce the distinguished color in the presence of catalysis substrates. The optical signal of the catalysis reaction, which is in a linear relationship with the initial template of Vp, could be determined with the naked eye or measured with Ultraviolet-visible (UV-vis) for qualitative and quantitative detections, respectively. Based on the optical signal intensity, rapid and easy detection of Vp was successfully achieved with satisfied sensitivity and specificity. Furthermore, the detection of *tdh*, *trh*, *tlh* and *toxR* virulence genes of two Vp species (Vp 33847 and Vp 17802) were all performed successfully with this developed colorimetric integrated PCR protocol, which demonstrated potential applicability for the rapid detection of other bacteria.

## 1. Introduction

The *Vibrio parahaemolyticus* pathogen is an important enteropathogen that causes acute human gastroenteritis throughout the world, and is an important seafood-borne pathogen globally [1]. As a Gram-negative and facultative halophilic bacterium, it is common in aquatic environments worldwide [2], including estuaries and seawater, and could be frequently isolated from zooplankton, coastal fish, and shellfish [3]. Due to its broad distribution, *V. parahemolyticus* (Vp) infections in China commonly occur in individuals living in coastal areas [4].

Detection of virulent strains in clinical and food samples using traditional culture methods could meet the practical requirements. Virulent strains exhibit no obvious growth phenotypes and could not be differentiated from non-virulent strains, and their populations are generally very small relative to other bacteria [5]. Therefore, we developed a simple, rapid, and label-free colorimetric method for *V. parahaemolyticus* (Vp) detection based on the platform of the traditional PCR. The target genes encoding virulence determinants and species-specific markers including *tdh*, *trh*, *tlh* and *toxR* were all adopted as the targets for virulent Vp strain detection [6]. These four specific gene markers have been positively used to identify Vp. [7]. For example, the pathogenesis of Vp has been associated with the production of the thermostable direct hemolysin (*tdh*) [8] and/or the thermostable direct-related hemolysin (*trh*) [9]. The thermostable direct hemolysin (*tdh*) and the thermostable-related hemolysin (*trh*) are the major recognized virulence factors. In addition, the *toxR* gene is initially described in *V. cholerae* as the regulatory gene for the cholera toxin and other virulence determinants, and was subsequently found in *V. parahaemolyticus*. The thermolabile hemolysin (*tlh*) gene is considered to be a useful target for the detection of *V. parahaemolyticus* because it was confirmed to exist in all of the *V. parahaemolyticus* stains identified to date.

Undoubtedly, polymerase chain reaction (PCR) has provided a revolutionary advance in the molecular diagnosis of nucleic acids because the process can be used to amplify trace amounts of a target DNA in an exponential manner. Amplified PCR products are typically detected by using agarose gel electrophoresis [10] or real-time methods employing fluorescent dyes [11]. However, these protocols are time-consuming or expensive and complicated instrumentation is required. Owing to these limitations, the development of alternative methods that can be employed for rapid and easy identification of PCR products without complicated instruments is of great significance. Extensive efforts targeted at this goal have led to new DNA sensing platforms that are based on molecular beacons [12] and nanoparticle-tagged probes [13,14]. However, these reported techniques require the use of fluorescence conjugation or probe thiolation procedures, both of which are costly and time-consuming [15,16,17,18,19,20]. Therefore, simple methods for the rapid identification of DNA analytes after PCR amplification without using complicated instruments remain in great demand. In this research, a new colorimetric method for rapid and easy detection of *Vibrio parahaemolyticus* was developed by the ingenious design of a primer for PCR amplification. A specific sequence forming the G-quadruplex DNAzymes under the special conditions was integrated with the common primer for *Vibrio parahaemolyticus* detection with PCR. The specific sequence for optical signal reporting was also amplified at the same time as target template amplification during PCR. The amplified sequence of the PCR product formed the mentioned G-quadruplex structure and bond with hemin mimicking the catalytic activity of peroxidase enzymes [21,22,23,24]. The optical signal intensity of the catalytic reaction was highly related to the original target DNA template of *V. parahaemolyticus*. Both rapid qualitative and quantitative detection of *V. parahaemolyticus* were successfully achieved based on naked-eye observation and UV-vis spectroscopic measurements of PCR products, respectively, which effectively broke the bottleneck of traditional PCR. Furthermore, four kinds of virulence genetic markers of two *V. parahaemolyticus* species were also detected successfully with our developed rapid detection strategy.

## 2. Experimental

### 2.1. Chemicals and Reagents

Hemin was purchased from Sigma-aldrich (St. Louis, MO, USA). Agarose was bought from Roche, Shanghai, China. Taq DNA polymerase, RNase, DNA Ladder (Dye plus), dNTPs (dATP, dGTP, dCTP, dCTP), TMB (3,3′,5,5′-tetramethylbenzidine) substrate reagent set and primer set designed for *V. parahaemolyticus* were all ordered from Sangon Biotechnology Co., Ltd. (Shanghai, China). *Salmonella typhimurium*, *Listeria monocytogenes*, *Staphylococcus aureus*, *Campylobacter jejuni*, *V. parahaemolyticus* 33847 (Vp 33847), *V. parahaemolyticus* 17802 (Vp 17802) were purchased from Prajna Biology Technique Ltd. (Shanghai, China). Ethylenedinitrilo)tetraacetic acid (EDTA), Tris (hydroxymethyl) aminomethane, SDS (Dodecyl sodium sulfate), PEG, NaCl and other reagents were obtained from Sinopharm Chemical Reagent Co., Ltd. (Shanghai, China). All other solvents and reagents used in the experiments were of analytical grade and used as received. Ultrapure water (Mill-Q, Millipore, Billerica, MA, USA, 18.2 MΩ resistivity) was used throughout this study.

### 2.2. Preparation of Magnetic Nanoparticles (MNPs) and Application in Genomic DNA Extraction of V. parahaemolyticus

Before the extraction of genomic DNA from bacteria, all bacteria were culture based on the instructions. Typically, 1 mL of *V. parahaemolyticus* maintained in glycerol broth at −80 °C, was inoculated into 5 mL of tryptone soy broth with 1% NaCl (TSBS) and incubated at 37 °C in a rotary shaker (200 rpm) for 12 h. Then 2 mL of the culture was transferred to 100 mL of TSBS and incubated at 37 °C in a rotary shaker (200 rpm) for 12 h. MNPs used in this research were synthesized and modified according to a previous reported method of our group. Briefly, 0.2 M FeCl_3_ (0.65 g) and 34 mM trisodium citrate (0.20 g) were completely dissolved in 20 mL ethylene glycol under vigorous stirring at room temperature in a nitrogen atmosphere. Next, NaAc (1.20 g) was added with vigorous stirring for 30 min. The mixture was sealed in a Teflon lined stainless steel autoclave and heated at 200 °C for 10 h, and then cooled down to room temperature. The carboxylic group functionalized MNP was centrifuged at 5000 rpm for 10 min, discarding the supernatant and washed with ethanol for three times. The collected MNPs were then re-dispersed in deionized water and stored at room temperature for further research.

SDS buffer (50 μL, 15%), Tris-HCl (50 μL, 1 M), proteinase k (50 μL, 20 mg/mL), EDTA (20 μL, 0.5 M, pH 5.0), deionized water (830 μL) and RNase (4 μL) were added into the tube in which the *V. parahaemolyticus* sample was first centrifuged at 10,000 rpm for 10 min at 4 °C and discarded the supernatant. The mixture was maintained at 70 °C for 10 min after vigorous vortexing. The solution was transferred to another clean tube in which PEG (100 μL, 9%), NaCl (500 μL, 5 M) solution and MNPs (20 μL) were contained. After culture for 10 min, the MNPs were collected with an applied magnetic field and rinsed three times with ethanol to elute the genomic DNA. Then the solution was dispersed in TE buffer (100 μL): Tris-HCl (10 mM), EDTA (1 mM, pH 8.0). The mixture was maintained at 65 °C for 10 min. The extracted DNA was analyzed by UV-vis spectrometry and agarose electrophoresis, respectively (detailed genomic DNA extraction results in Supplementary Materials).

### 2.3. PCR Amplification of V. parahaemolyticus

Extracted DNA of *V. parahaemolyticus* (Vp 33847 and Vp 17802) were directly used as template for PCR amplification with our designed specific primer set. All detailed sequence information of primer sets were in Table S1. In each case, PCR was performed in a total volume of 25 μL containing 2.5 μL DNA template, 2.5 μL 10× buffer, 2.5 μL 20 mM MgCl_2_, 200 μM each dNTP, 1 μM of each primer, and 1.25U Taq DNA polymerase. PCR was carried out under the following conditions: denaturation at 94 °C for 3 min, followed by 30 cycles of denaturation at 94 °C for 1 min, annealing at 58 °C for 1 min, and primer extension at 72 °C for 1 min. A final extension was performed at 72 °C for 5 min. The concentration of PCR product was determined by measuring the absorbance at 260 nm using a UV-vis spectrometer. DNA amplification was also confirmed by using electrophoresis in 2% agarose gel. The PCR control was performed in the absence of target DNA (without the bacterial colony). The specificity and generality of the designed primer set of PCR was assessed using DNA from different non-target bacterial colonies (*Salmonella typhimurium*, *Listeria monocytogenes*, *Staphylococcus aureus*, and *Campylobacter jejuni*).

### 2.4. Colorimetric Detection of V. parahaemolyticus (33847 and 17802)

The color of the PCR product was adopted as the criterion for qualitative and quantitative detection of *V. parahaemolyticus* by naked-eye and UV-vis, respectively. Briefly, 50 μL PCR products were added into the mixture including 4 μL KCl (500 mM), 8 μL HEPES buffer, 4 μL hemin (50 μM) and the solution was incubated at 37 °C for 15 min. Then, 88 μL of freshly prepared TMB reagent was quickly added to the above mentioned mixture and incubated at room temperature for 10 min. When the color of reaction mixture turned to blue, the results could be qualitatively determined by naked-eye or recorded the results with digital camera. After addition of 50 μL H_2_SO_4_ (0.2 M) for termination of reaction, colorimetric measurements at 450 nm were performed by using a microplate scanning spectrometer for quantitative detection.

## 3. Results and Discussion

### 3.1. Design of the Rapid Colorimetric Strategy for Rapid and Easy Detection of V. parahaemolyticus

**Scheme 1:**
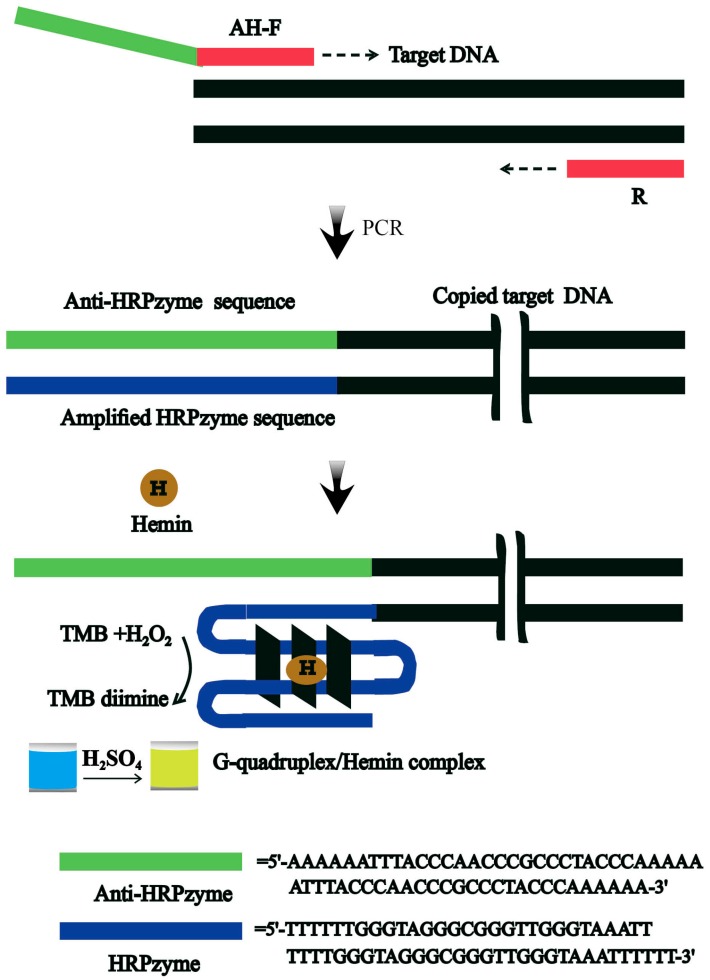
The schematic diagram of the colorimetric integrated PCR protocol for rapid detection of *V. parahaemolyticus* (R: reverse primer; AH-F: forward primer).

The strategy we developed for the colorimetric detection of *V. parahaemolyticus* is outlined in Scheme 1. Firstly and importantly, a specific forward primer as shown in Scheme 1 was designed to contain three regions comprised of a sequence that is complementary to that of the target template DNA of *V. parahaemolyticus*, with a segment of poly A sequence as a linker, and a specific sequence (anti-HRPzyme) that is complementary to the HRPzyme for signal reporting. By using these forward and normal reverse primers in the presence of the target DNA of *V. parahaemolyticus*, the amplified double-strand PCR products containing the HRPzyme sequence existed in a great amount. These special PCR products could catalyze the oxidation of TMB by the formation of the G-quadruplex/hemin complex in the presence of hemin, generating the distinguished blue catalyzed products. The optical signal intensity of the catalysis system could be determined or measured with the naked eye or the UV-vis spectrometer, respectively.

### 3.2. The Feasibility Study of the Designed Colorimetric Method

**Figure 1:**
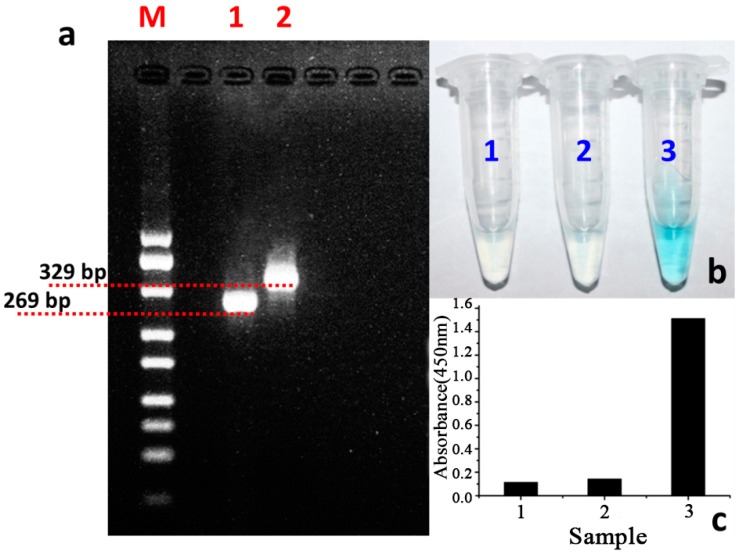
The possibility confirmation results of the developed colorimetric integrated PCR protocol for rapid and easy detection of *V. parahaemolyticus*. (**a**) The agarose gel electrophoresis results of the PCR products with traditional and designed primer sets, M: marker; lane 1: PCR products with traditional primer set; lane 2: PCR product with our designed primer set; (**b**) Colorimetric detection results of *V. parahaemolyticus*, sample 1: negative control without template in PCR; sample 2: PCR product with traditional primer set; sample 3: PCR product with our designed primer set; (**c**) Quantitative results of (**b**).

Firstly, we tested the feasibility of the developed strategy for the rapid and easy colorimetric detection of *V. parahaemolyticus*. The designed sequence of the primer for PCR amplification was compared with that of the non-designed primers. As the agarose gel electrophoresis results show in Figure 1a, both the designed primer set and the traditional set could be successfully applied in the specific amplification of the target sequence of Vp. More importantly, compared with the standard marker in lane 1, the amplification product in lane 3 is about 60 bp larger than that in lane 2, which is the amplification product of the traditional primer set. This difference of the PCR products between lane 2 and 3 could be attributed to the specific integrated sequence of the HRPzyme sequence of 60 bp. In addition, this validates that the designed specific primer set could be well applied in the detection of target *V. parahaemolyticus*. Furthermore, the signal reporting property of the designed protocol was also confirmed. Results in Figure 1b demonstrated that, compared with the negative control (sample 1 in Figure 1b), the PCR product using the traditional primer set could not induce the color change of the solution (sample 2 in Figure 1b) while the PCR product based on the designed primer set could induce the obvious color change of the reaction system (sample 3 in Figure 1b). The quantitative analysis of the different reaction system was also carried out and shown in Figure 1c, which further confirmed the signal reporting possibility of our designed detection protocols. In short, based on the designed functional primer set, the product of traditional PCR could be effectively measured in the colorimetric model.

### 3.3. Optimization of the Detection Conditions

Following, we adopted the designed colorimetric protocols for target *V. parahaemolyticus* detections. In addition, some key factors were optimized firstly for achieving the best sensing performance. Of note, the optical intensity of the catalyzed reactions of PCR products under different conditions was subtracted from the control and analyzed with the others for the best signal response and sensing performance.

**Figure 2:**
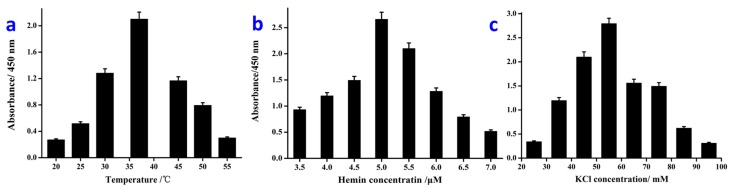
Condition optimization results for rapid colorimetric detection of *V. parahaemolyticus*. (**a**) Temperature optimization results; (**b**) Optimization of the hemin concentrations; (**c**) Optimization of KCl concentrations for coloration of the catalysis reaction.

Firstly, the incubation temperature of the catalytic coloration reaction was studied. From the results shown in Figure 2a, it is easy to find that the strongest optical signal intensity occurs at 37 °C, which is defined as the optimal temperature for following the sensing research. Similarly, the optimal concentration of hemin in the catalysis system was found to be 5 μM as shown in Figure 2b. Besides, for the HRPzyme-based catalysis reaction, the concentration of KCl is of great importance for the catalysis efficiency. It is also demonstrated that the best concentration of KCl is 55 mM, at which the maximum optical density induced by the HRPzyme catalysis could be achieved (Figure 2c). One thing should be pointed out: in our research, the colorimetric analysis is carried out directly based on the dsDNA of PCR products, and the colorimetric signal is slightly reduced compared with that of the ssDNA HRPzyme. After optimization, the colorimetric results could well meet the requirements for rapid qualitative and quantitative detection of target bacterium.

### 3.4. Colorimetric Detection of Target V. parahaemolyticus

**Figure 3:**
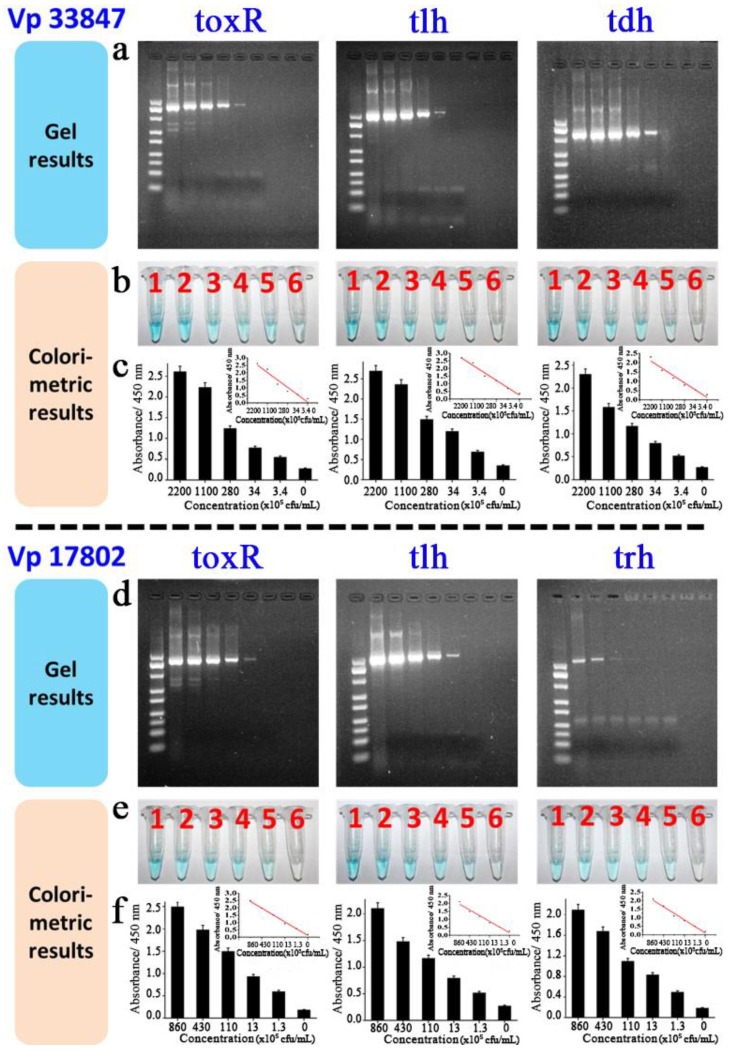
Colorimetric detection results of *V. parahaemolyticus* 33847 and 17802. For *V. parahaemolyticus* 33847 detections, both the gel electrophoresis (**a**) and colorimetric methods (**b**) were carried out at the concentrations of 2200 × 10^5^, 1100 × 10^5^, 280 × 10^5^, 34 × 10^5^, 3.4 × 10^5^ cfu/mL, respectively; Absorbance (**c**), three virulence genes including *toxR* 33847, *tlh* 33847 and *tdh* 33847 were detected with our proposed colorimetric method; For *V. parahaemolyticus* 17802 detections, both the gel electrophoresis (**d**) and colorimetric methods (**e**) were carried out at the concentrations of 860 × 10^5^, 430 × 10^5^, 110 × 10^5^, 13 × 10^5^, 1.3 × 10^5^ cfu/mL, respectively; Absorbance (**f**), three virulence genes including *toxR* 17802, *tlh* 17802 and *trh* 33847 were detected with our proposed colorimetric method; Sample 6 in both detections was the negative control.

In our research, the final colorimetric analysis of the virulence genes of different Vp species is directly based on the PCR amplification. In addition, the genomic DNA samples were extracted with the magnetic nanoparticles according to our previously reported methods. Firstly, the amplification of four virulence genes (*toxR*, *tlh*, *tdh* and *trh*) of two Vp species (Vp 33847 and 17802) at different concentrations was measured with both agarose gel electrophoresis and the colorimetric method. From the agarose gel electrophoresis results shown in Figure 3a,d, firstly, it is easy to see that the three virulence genes of Vp 33847 (*toxR* 33847, *tlh* 33847 and *tdh* 33847) and Vp 17802 (*toxR* 17802, *tlh* 17802 and *trh* 17802) were all successfully amplified at different concentrations. Following, all these PCR-amplified products were measured with our developed colorimetric method by directly adding them into the catalysis system. The qualitative results shown in Figure 3b and e indicate that the PCR products could induce the color change of the solution based on the HRPzyme-like catalytic property of the G-quadruplex/hemin complex compared to all negative controls without the template in PCR (sample 6 in all groups in Figure 3b,e). All these optical signals could be observed and distinguished from the negative controls with the naked eye. Furthermore, for quantitative analysis, the linear relationship was constructed between the optical intensity and the concentration of the target *V. parahaemolyticus*. As shown in Figure 3c,f, all detections of the virulence genes of two *V. parahaemolyticus* species have a good linear relationship with little variation in slope. In addition, the best limit of detection could be as low as 3.1 × 10^5^ and 2.9 × 10^4^ cfu/mL for *V. parahaemolyticus* 33847 and 17802, respectively, according to the 3σ rules. These quantitative results of our colorimetric method are comparable to gel electrophoresis-based traditional PCR or other methods and are about 10-fold better than classic culture-based methods, demonstrating the potential practical usage in rapid and sensitive detection of various analytes based on the principle of traditional PCR.

**Figure 4:**
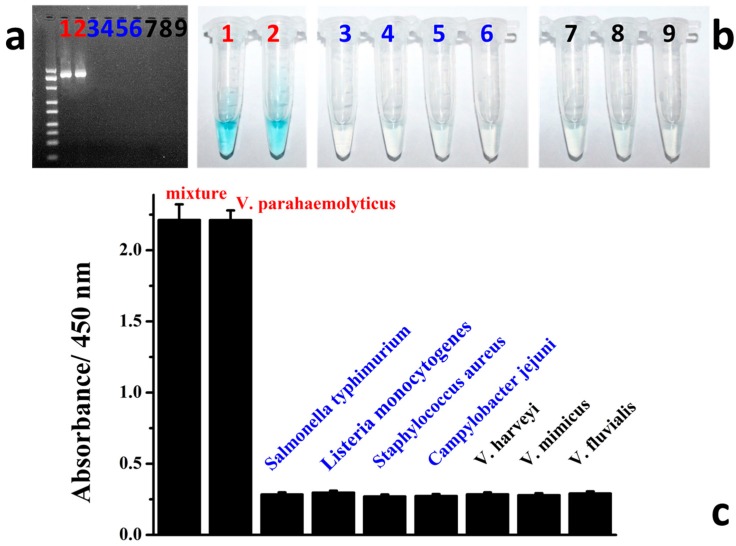
Specificity integration results of *V. parahaemolyticus* detections. Based on the specific designed primer set, both the gel electrophoresis and colorimetric methods demonstrated excellent specificity for *V. parahaemolyticus* detections. Agarose gel analysis (**a**) of amplified PCR products and colorimetric response (**b**), absorbance (**c**).

Furthermore, the specificity of our designed protocol for rapid detection of *Vibrio parahaemolyticus* was tested and the results are shown in Figure 4. Nine kinds of samples including the mixtures of several bacteria, *V. parahaemolyticus*, *V. harveyi*, *V. mimicus*, *V. fluvialis*, *Salmonella typhimurium*, *Listeria monocytogenes*, *Staphylococcus aureus*, and *Campylobacter jejuni*, were chosen based on the previous studies [25,26,27,28,29] and detected using our protocol, respectively. From the results in Figure 4a, it could be found that only the samples of the mixture and *V. parahaemolyticus* could be amplified with the target band with the gel electrophoresis while other non-target samples could not be amplified. The colorimetric operations were further confirmed in the same procedures as mentioned above. As expected, only the PCR products of the mixtures and *V. parahaemolyticus* group could induce the obvious optical signal of the blue color while other groups could not (Figure 4b,c). All these results indicated that, with the designed functional primer set, the colorimetric protocol could be applied in the rapid and easy detection of *V. parahaemolyticus* with high specificity without any interference.

## 4. Conclusions

In summary, a new HRPzyme-based catalytic colorimetric method was developed for the rapid and easy detection of *V. parahaemolyticus*. A segment of the functional sequence was integrated with the primer and amplified with PCR. This designed functional sequence in the PCR product induced the obvious occurrence of the optical signal, which could be distinguished or measured by the naked eye or the UV-vis spectrometer, respectively. Four characteristic target genes of *Vibrio parahaemolyticus* including *toxR*, *tlh*, *trh* and *tdh* were successfully determined based on the colorimetric signals. Of greater significance, this designed colorimetric protocol could be adopted for the rapid and easy detection of any other pathogens by integrating the unique functional sequence with the traditional primer set for producing signals. With further research in sensitivity improvement, this colorimetric method would greatly facilitate the on-site detections or routine screenings in resource-limited fields.

## Supplementary Materials

The supplementary materials are available online at http://www.mdpi.com/1424-8220/16/10/1600/s1.
