# Supplementary material for: Colorimetric Integrated PCR Protocol for Rapid Detection of Vibrio parahaemolyticus
Source: Sensors (Basel). 2016 Sep 28;16(10):1600. doi: 10.3390/s16101600 (PMC5087389; doi:10.3390/s16101600)
Supplement: Supplementary file 1 [file sensors-16-01600-s001.pdf]

Supplementary Materials: Colorimetric Integrated PCR Protocol for Rapid Detection of *Vibrio parahaemolyticus*

Kewen Cheng, Daodong Pan, Jun Teng, Li Yao, Yingwang Ye, Feng Xue, Fan Xia and Wei Chen

Table S1. Oligonucleotide primers used in this work.

Species	Taget	Primers (5′-3′)	Amplicon Size (bp)
V. parahaemolyticus	toxR	GTCTTCTGACGCAATCGTTG<br>ATACGAGTGGTTGCTGTCATG	366
V. parahaemolyticus	Tlh	AAAGCGGATTATGCAGAAGCACTG<br>GCTACT TTCTAGCATTTTCTCTGC	450
V. parahaemolyticus	Trh	TTGGCTTCGATATTTTCAGTATCT<br>CATAACAAACATATGCCCATTTCCG	500
V. parahaemolyticus	Tdh	GTAAAGGTCTCTGACTTTTGGAC<br>TGGAATAGAACCTTCATCTTCACC	269
Salmonella spp.	orgC	CTTTATGATGCATTCTACCAACGACTG<br>CCGAATCACCACTGTTAGGA	121
L. monocytogenes	hlyA	ACTTCGGCGCAATCAGTGA<br>TTGCAACTGCTCTTTAGTAACAGCTT	137
S. aureus	entA	AAGTGCCGATCAATTTATGGCTA<br>CCTGAACAGTTACATTTTTCTTATTCGT	90
C. jejuni	cfrA	AGCAGGGATAAGCCCTCTTG<br>AGCGATCTATTTGCCAYTCG	203

In front of the forward primer plus the following sequence: AAAAAATTTACCCAACCCGCCCTA CCCAAAAAATTTACCCAACCCGCCCTACCCAAAAAA.

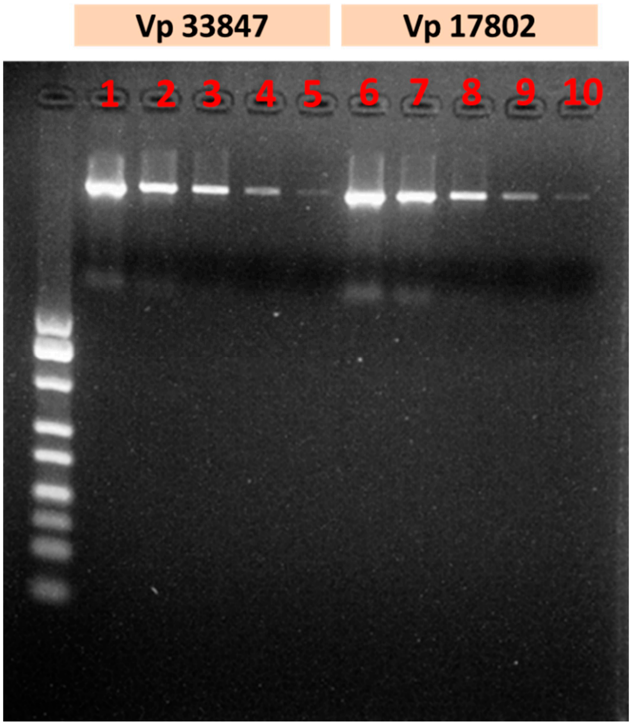

Figure S1. The agarose gel electrophoresis results of the extracted genomic DNA from *V. parahaemolyticus* 33847 and 17802. Lane 1–5: the extracted genomic DNA from *V. parahaemolyticus* 33847 with the dilution of 1-, 2-, 8-, 64- and 640-folds; Lane 6–10: the extracted genomic DNA from *V. parahaemolyticus* 17802. The ladder of DNA marker is 500 bp.
